# The introduction and spread of rye (*Secale cereale*) in the Iberian Peninsula

**DOI:** 10.1371/journal.pone.0284222

**Published:** 2023-05-10

**Authors:** Luís Seabra, Andrés Teira-Brión, Inés López-Dóriga, María Martín-Seijo, Rubim Almeida, João Pedro Tereso

**Affiliations:** 1 InBIO Associate Laboratory, CIBIO, Research Center In Biodiversity and Genetic Resources, University of Porto, Vila do Conde, Porto, Portugal; 2 BIOPOLIS Program in Genomics, Biodiversity and Land Planning, CIBIO, Vila do Conde, Porto, Portugal; 3 Faculty of Sciences, Department of Biology, University of Porto, Porto, Portugal; 4 School of Archaeology, University of Oxford, Oxford, Oxfordshire, United Kingdom; 5 Wessex Archaeology, Salisbury, Wiltshire, United Kingdom; 6 Departamento de Ciencias Históricas, Universidad de Cantabria, Santander, Cantabria, Spain; 7 MHNC—UP—Natural History and Science Museum of the University of Porto, Porto, Portugal; 8 Centre of Interdisciplinary Studies of Coimbra, Coimbra, Portugal; 9 Centre for Archaeology, UNIARQ, School of Arts and Humanities, University of Lisbon, Lisbon, Portugal; Austrian Academy of Sciences, AUSTRIA

## Abstract

Some of the earliest Western European macroremains of rye (*Secale cereale*) have been recently recovered in Northwest Iberia. However, the chronological and cultural contexts of these remains have not been yet exhaustively analysed. To address this gap of research, previous and unpublished assemblages have been reviewed and analysed through an analytical set of methods: biometry, radiocarbon dates and integrating the remains of rye in the broad archaeobotanical record of the region. Results show the earliest macroremains of rye in the Iberian Peninsula date to a period between the 3^rd^ century and the first half of the 1^st^ century BCE. Rye was usually found in assemblages dominated by spelt and other cereals, in whose fields it was likely acting as a weed. There is no record of rye for about the two following centuries, after which it is probably reintroduced, now as a crop. It is found in several sites from the 3^rd^-4^th^ centuries CE onwards, suggesting it is a staple crop as in other regions in Europe. Significant differences in grain size are only recorded in a 10^th^-11^th^ century settlement, suggesting few changes in grain morphometry before Medieval times.

## Introduction

Rye (*Secale cereale*) did not travel as part of the agricultural crop package that spread from Southwest Asia to the Atlantic during the Neolithic [1–3]. Conversely, its history in the European continent is more complex than other major cereals such as wheat or barley. Rye encompasses different primary and secondary domestications and a diversity of expansion/constrain events [1–3], which results in a biography still poorly known, but at the same time, an intricate and exciting challenge yet to be deciphered holistically.

The cross-pollination of the genus *Secale* favoured wide genetic diversity and frequent hybridization, originating a long-lasting dispute about rye taxonomy and some uncertainty concerning the ancestry of the domesticated species (e.g. [4,5]). Still, up-to-date genomic analyses suggest *Secale cereale* subsp. *vavilovii* was its wild ancestor and it is naturally distributed through Southwest Asia, where the earliest macroremains of rye have been found (e.g. [2,6,7]).

From an archaeobotanical point of view, the differentiation of wild and cultivated rye in absence of chaff is problematic [8–10], in addition to the difficulty of distinguishing between wild rye and wild einkorn [11–13]. Furthermore, the weedy/cultivated character of early findings has been a matter of debate (e.g. [1,2,14]). As such, even when remains of rye were found outside the natural range of its wild populations, it was unclear if the species was in fact being deliberately cultivated. Plus, *Dasypyrum villosum* further complicates this issue since it shows similar morphological characteristics [9,15,16]. In fact, studies including the re-examination of Bronze and Iron Age material have shown the resemblance among these cereals, and that possible misidentifications could have occurred [15,16].

The separation between pollen of wild and domesticated rye is also not possible [1,17]. Still, the fact that rye pollen morphology differs from the remaining domesticated cereals and rye spikes generate high pollen quantities, which can reach long distances, may allow pertinent information for archaeobotanical studies, even though with due caution (see discussion below) (e.g. [1,17–21]).

The systematization carried out by Behre [1] is a landmark in the subject and is still the main reference regarding the history of rye cultivation in Europe. From Southwest Asia, rye would have migrated into Europe through the Balkans, as suggested by the several Neolithic findings in south-eastern sites [2,22,23]. Still, macroremains of rye are rare in Neolithic contexts in Europe and even during the Bronze Age, as such, they are usually interpreted as weeds [1,2,22,24]. Based on its increasing presence in Iron Age contexts and regional agricultural changes, some authors suggest the cultivation of rye began during this period in Central and Eastern Europe [1,22]. Although, in the Baltics, it likely occurred only in the 2^nd^–3^rd^ centuries CE [17]. Still, the timing and social context of rye´s introduction and expansion in western Europe remain poorly known. In the British Isles, rye was recovered in reduced amounts in Late Iron Age and Roman contexts and its intentional cultivation was not certain, although it could have been a minor crop during the last period [25]. As far as the Iberian Peninsula is concerned, almost no information is available for the Pre-Roman and Early Roman times.

## Rye in Iberia

### The pollen evidence

Pollen assemblages from natural deposits and archaeological sites show the presence of *Secale* in the Iberian Peninsula in multiple timespans. Some of these assemblages document the presence of few, sometimes single pollen grains of *Secale* in prehistoric levels. That is the case of Planell de Perafita (Andorra, Northeast Iberia) where a single pollen grain was found in a context chronologically attributed to the Neolithic [26,27]. Nearby, a few grains were equally found in Mesolithic and Neolithic levels at Lake Racout (French Pyrenees) [27,28]. *Secale*-type pollen was sporadic in Southwestern Iberia, namely in the sites of Barbaroxa de Baixo (Setúbal) and Lagoa Travessa I (Setúbal), in Neolithic contexts [27,29,30]. Numerous palynological studies have been carried out in Northwest Iberia (e.g. [31–37]), but *Secale* pollen has been barely found. The earliest evidence came from Chan do Lamoso (Lugo), where scarce remains were recorded in samples with chronologies between the Late Neolithic/Chalcolithic and the Early Bronze Age [37]. However, the possibility of such early microremains may correspond to *Lygeum spartum* was admitted [37].

*Secale*, *Secale*-type, and *Secale cereale* pollen grains are recurrent in Bronze and Iron Age deposits, appearing in more sites and Iberian areas than in the previous periods (e.g. [29,30,37–43]). Still, the general pattern is identical, with the exception of the record from Chan de Lamoso, which suggests a more consistency of *Secale* pollen from the Middle Bronze Age onwards, although discontinuous events were equally noted [37]. Identifications at the species level (*Secale cereale*) were proposed in Late Bronze and Iron Age contexts from Central Iberia and in one Iron Age deposit in Northwest Iberia, but pollen grains have been recovered in small quantities [41,42,44].

The record in the Roman period is somehow vague, being rye pollen present in a few sites [29,30,41,45–47]. The assemblage from Cánãr (Granada, Southern Iberia) seems more significant since it shows its recurrent presence, which inclusively increases in number in later phases [47]. Pollen data suggest rye is more frequent in Late Antiquity, and above all, in the Medieval period when it is found in several cores and throughout the Iberian territory (e.g. [26,37,38,45–52]). Globally, in this last period, rye pollen continues to be present in reduced amounts, but significant assemblages are observed in some cases (e.g. [47–49,51]).

### Grains and chaff of rye in the archaeological record

The early history of rye cultivation in the Iberian Peninsula has not been addressed in a systematic way, though since long, it has been identified in several Late Antique and Medieval sites (S1 Table). The earliest macrobotanical findings of rye from Northeast Iberia were recorded in Late Antique contexts from sites such as Carrer dels Bafart (Lleida) and Vilauba (Girona) [53–55], and in the surrounding areas, such as Andorra or Aragon. Still, in most sites rye was scarce [54–64]. In other Northern areas as well as in Central and Southern Iberia, rye was seldom found. Around 150 grains were collected in Medieval contexts (10^th^–12^th^ centuries CE) at Gasteiz (Álava) [65] and at the Visigothic (6^th^–8^th^ centuries CE) occupation of Gózquez (Madrid) even fewer grains were recovered [66]. Rye has also been recorded in Islamic contexts (9^th^-13^th^ centuries CE) at Melque (Toledo), Albalat (Cáceres) [67,68] and Castelo de Silves (Faro) [69].

Until a few years ago, the scenario was similar for Northwestern and Western regions. At Monte Mozinho (Porto, Northern Portugal), grains and chaff of rye were recovered from a Late Roman deposit (3^rd^–4^th^ centuries CE), in agreement with the Catalonian sites [70]. The northernmost remains, from Galicia, dated from Medieval times (13^th^–15^th^ centuries CE) [71–73]. All changed after the archaeobotanical study carried out at Crastoeiro (Vila Real, Northern Portugal). Grains and chaff of rye were identified, and the ^14^C dates obtained pointed out a chronology around the 1^st^ century BCE [74]. At the time, these were the earliest securely dated macroremains of rye in the whole Iberia.

**Fig 1:**
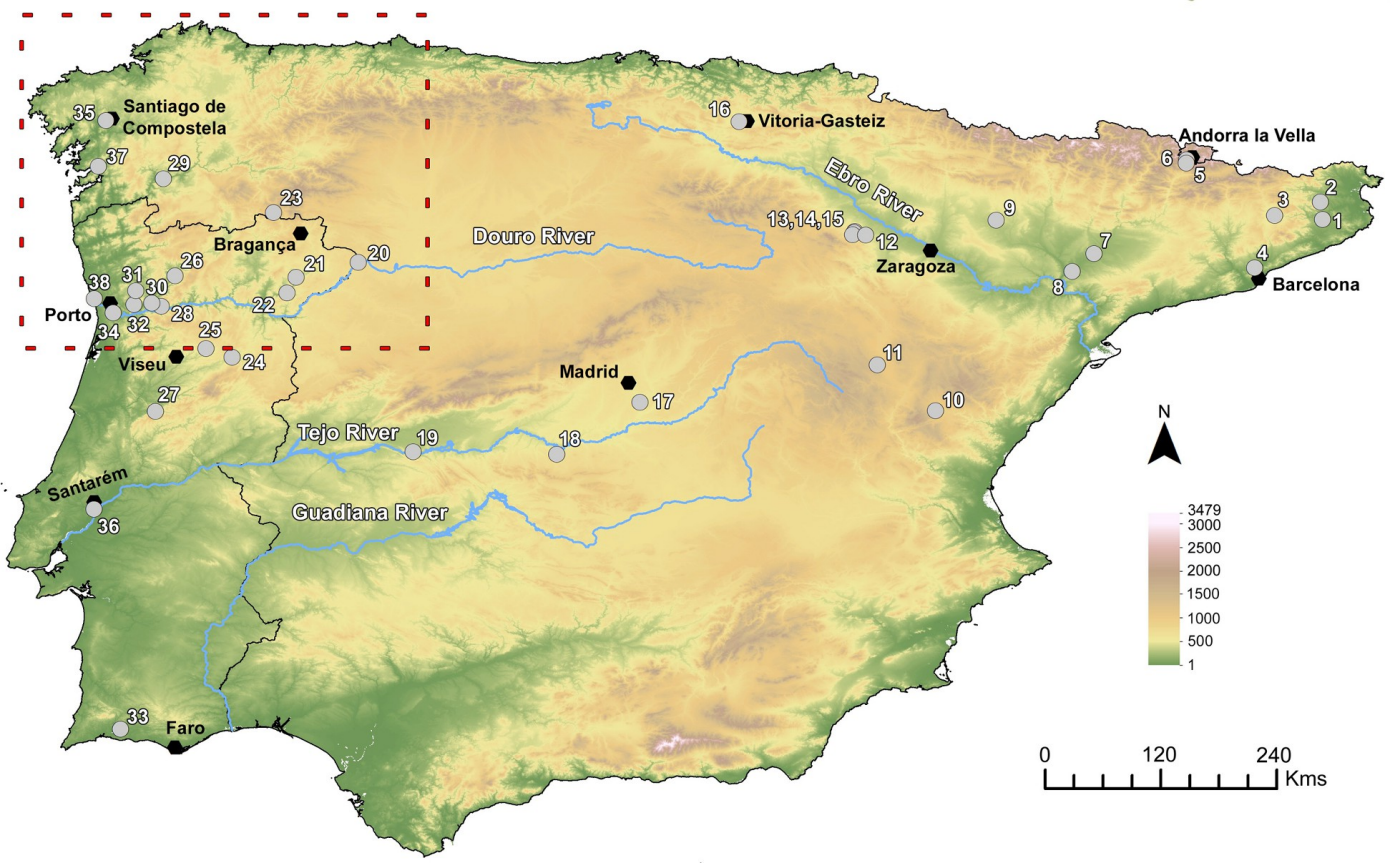
Iberian sites with macroremains of rye until the Late Medieval period (15^th^ century CE) (see more details in S1 Table). Legend: 1-Can Gelats, 2-Vilauba, 3- l´Esquerda, 4-Els Mallols, 5-Camp Vermell, 6-Roc d´Enclar, 7-Carrer dels Bafart, 8-Bovalar, 9-Las Sillas, 10-El Quemao, 11-Cabezo de la Cisterna, 12-Los Pozos de Bureta, 13-La Mora Encantada, 14-Palacio de Bulbuente, 15-Casa Conventual de Ambel, 16-Gasteiz, 17-Gózquez, 18-Melque, 19-Albalat, 20-Castro S. João das Arribas, 21-Cabeço da Grincha, 22-Quinta de Crestelos, 23-Castelo Pequeno de Santigoso, 24-São Gens, 25-Senhora do Barrocal, 26-Crastoeiro, 27-Quinta da Torrinha, 28-Cruito, 29-Castro de Astariz, 30-Freixo/Tongobriga, 31-“Casa Romana” (Roman house) of the Castro de São Domingos, 32-Monte Mozinho, 33-Castelo de Silves, 34-Castelo de Crestuma, 35-A Rocha Forte, 36-Convento de S. Francisco de Santarém, 37-Ponte do Burgo, 38-Castro de Guifões. This figure includes European Digital Elevation Model datasets from the Copernicus Land Monitoring Service [75].

Since then, several archaeobotanical approaches have been developed, addressing sites with different characteristics and chronologies, and remains of rye, both grains and chaff, were recovered more frequently (Fig 1). This study will explore recent and unpublished data in order to: 1) determine the chronology of rye introduction and dissemination, focusing on Northwest Iberia; 2) discuss the domesticated/wild status of the recorded remains; 3) document eventual changes in grain size through time, and 4) assess the social and environmental context of rye farming until the Medieval Period.

## Materials and methods

### Selection of sites

The study focused on rye macroremains recovered from pre-Medieval and Medieval sites. Although written documentation points to a widespread cultivation of rye in Medieval times [76,77], concrete information on its introduction and its early expansion is lacking. An extensive research was carried out to obtain an updated overview of rye cultivation. As a result, 15 sites with remains of rye dating from the Iron Age to the Medieval period were encompassed in the framework of this study, namely: “Casa Romana” (Roman house) of the Castro de São Domingos (CDCR), Castelo Pequeno de Santigoso (CPS), Crastoeiro (CRT), Cruito (CRUI), Freixo/Tongobriga (TONG), Castro de Guifões (GUIF), Cabeço da Grincha (CDG), Castro de Astariz (AST), Monte Mozinho (MOZ), Crestuma (CRES), Castro S. João das Arribas (CSJAMD), Quinta de Crestelos (QCREST), Senhora do Barrocal (SB), Ponte do Burgo (PDB), A Rocha Forte (ARF). Fruits/seeds assemblages with rye were analysed to identify whether rye was a dominant or secondary cereal (see S2 Table). Quantification of remains only took into consideration whole or fragmented grains with embryo cavity and rachis fragments (nodes and internodes). No permits were required for the described study, which complied with all relevant regulations.

Remains of rye were recorded in other sites in Northwestern Iberia but they were not taken into consideration in this study for a series of reasons. At Cortegada (Silleda, Northwest Spain), Arnanz and Chamorro [78] allegedly identified rye in a preliminary report from an Iron Age hillfort, but there were no pictures of the remains or detailed reference to the archaeological contexts where they were retrieved [79]. The existence of rye at Castro de Vilela (Taboada, Northwest Spain) has been mentioned by the team who excavated the site [80], without mentioning any archaeobotanical work or radiocarbon dates. At Alto do Coto da Pena (Caminha, Northwest Portugal), grains of rye identified by A.R. Pinto da Silva [81] were mistakenly attributed to the Bronze Age [82]. A careful look at the original labels that accompany the remains demonstrated us that the grains were recovered in C1 (layer 1). According to the chief archaeologist [83], and personal communication) layer 1 is a top level dating to the Medieval period. The findings from A Franqueira (A Cañiza, Northwest Spain) [84] were considered unreliable in previous investigations [85]. Lastly, the several grains found inside Iron Age vessels at Alcáçova de Santarém (Santarém, Central Portugal), identified as rye by Queiroz et al. [86] have now been determined as barley grains after a careful look at the photos provided in the publication and a new laboratory assessment.

### Chronology

The chronology attributed to the macroremains of rye in the referred publications was recorded but whenever possible it was confirmed with direct ^14^C dates. Radiocarbon dates were obtained on 18 grains of rye from 10 of the 15 reviewed sites: CDCR (n = 1), CRT (n = 2), CPS (n = 1), TONG (n = 3), CRUI (n = 1), CDG (n = 1), MOZ (n = 2), CRES (n = 5), CSJAMD (n = 1), SB (n = 1). General information about these latter sites and the analysed archaeological contexts, as well as other data concerning the composition of the fruits/seeds assemblages, radiocarbon dates, and measured grains, are individually exposed in supporting information (S1 Text).

A Bayesian analysis including Kernel Density Plots (KDE_Plot) was carried out to obtain a more detailed chronological framework about the chronology of rye, particularly concerning its earliest appearance and presence through time in Northwest Iberia. For such, the OxCal software v.4.4.4 [87] was used, applying one sequential model with two phases (here replaced by the OxCal KDE_Plot command with the same function), following Bronk Ramsey [88,89]. Adding the KDE_Plot function allows an overview of the temporal distribution of the dates in each phase [89].

The separation of the ^14^C dates into two groups was due to the time interval observed between the Iron Age/Early Roman and Roman calibrated dates. In order to confirm and detail this alleged gap, the OxCal Interval command was applied in the model [88]. The lack of coherent stratigraphic data and the complexity of dealing with multiple sites led us to include the ^14^C dates within each group according to their relative chronological order. This process was conducted previously to the model creation, using the OxCal Order command [88]. Uniform boundaries were applied due to this uncertainty about the order of the dates [88]. The full OxCal code (CQL2) inserted is available in supporting information (S2 Text).

### Biometry

Only well-preserved grains of rye were characterized, thus permitting the measurement of length, width, and thickness. The biometric characterization was conducted on a minimum of 50 individuals per site and up to 100 individuals whenever possible. Grains were measured using a digital calliper with an accuracy of ± 0.02 mm (Table 1). Biometric variables and ratios were compared across sites using one-way ANOVA. Association between relevant pairs of variables was assessed using correlation analysis. A level of significance of 0.05 was used in both analyses. A Principal Component Analysis (PCA) was conducted to extract the main gradients of the morphological profile of the samples. All analyses were carried out in R (version 4.1.3; [90]) using the IDE RStudio (version 2022.2.1.461; [91]). Data visualization was also performed in R and figures were built with plots produced using packages “ggplot2” [92], “ggord” [93], “envalysis” [94], and “ggpubr” [95] (S1 Fig).

**Table 1:** Rye grains measured (provenance and amounts).

Site	Stratigraphic unit (S.U.)	Context	Period	Measured grains (N°)
CRT	18c	Pit 18	Iron Age/Early Roman	3
	18d			3
	18.1a	Pit 18.1		5
	18.2	Pit 18.2		45
	18.5	Pit 18.5		1
CPS	204	Utilization layer	Iron Age	100
MOZ	6	Quad. structure	Late Roman	14
	78	Destruction layer	Late Antiquity	59
	204	Oven		27
CSJAMD	225	Destruction layer	Late Antiquity	100
SB	107	Fire level	Medieval	100

## Results

### Rye in the archaeobotanical record

**Fig 2:**
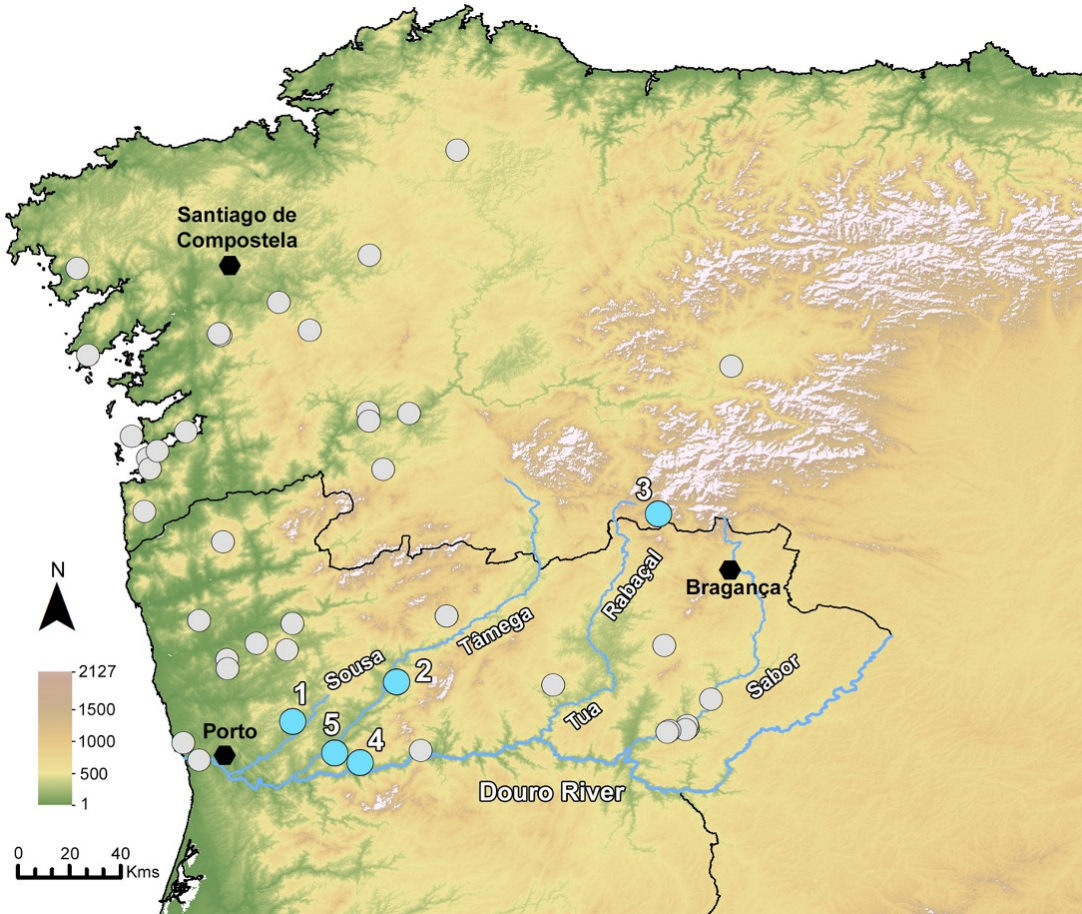
Fruits/seeds assemblages with (blue dots) and without (grey dots) rye from Iron Age and Early Roman contexts (4^th^ century BCE-1^st^ century CE) in Northwest Iberia. Legend of sites with rye: 1- CDCR, 2- CRT, 3- CPS, 4- CRUI, 5- TONG. This figures includes European Digital Elevation Model datasets from the Copernicus Land Monitoring Service [75].

**Fig 3:**
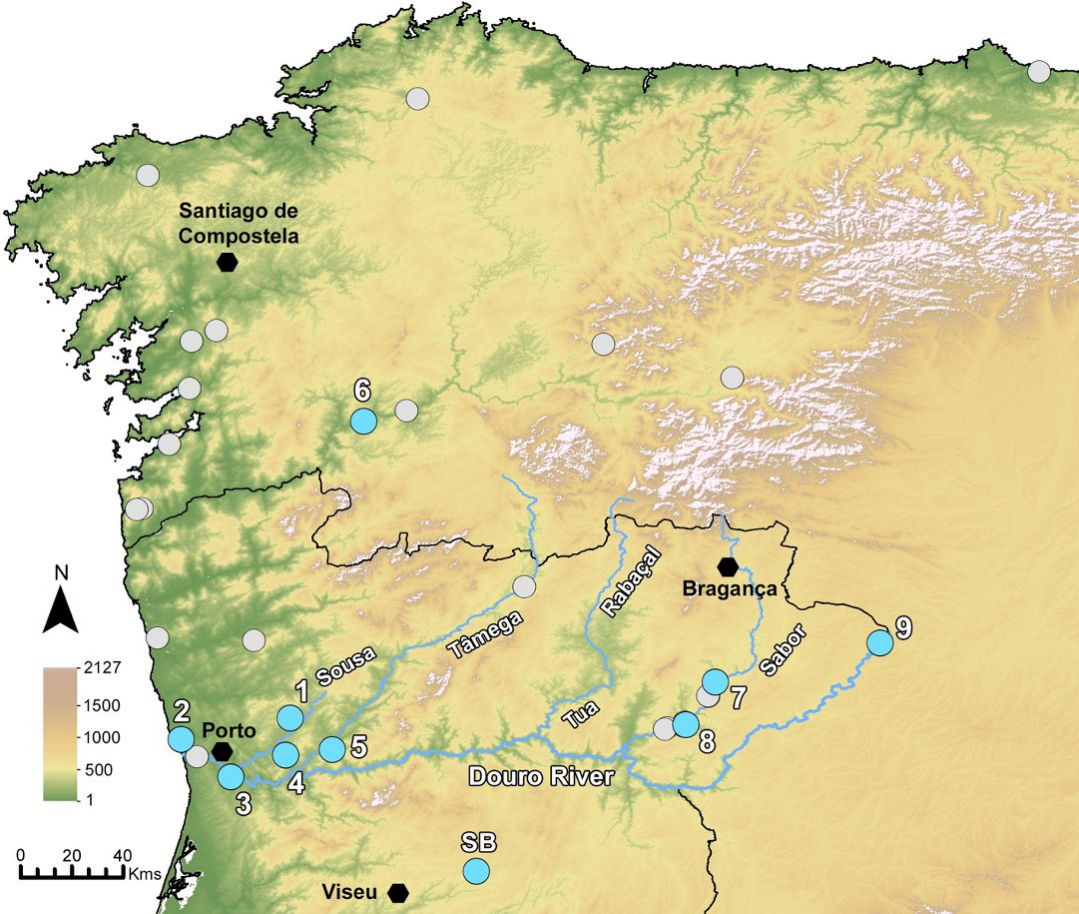
Fruits/seeds assemblages with (blue dots) and without (grey dots) rye from Roman and Late Antique contexts (2^nd^ century CE-7^th^ century CE) in Northwest Iberia. Legend of sites with rye: 1- CDCR, 2 –GUIF, 3- CRES, 4- MOZ, 5- TONG, 6- AST, 7- CDG, 8-QCREST, 9- CSJAMD. The only medieval site with a ^14^C date of rye (SB) was also included in this figure. This figures includes European Digital Elevation Model datasets from the Copernicus Land Monitoring Service [75].

Rye was found in 14 sites in Northwestern Iberia, most of them located in the Douro basin (see Figs 2 and 3 and S2 Table). Senhora do Barrocal (SB), although located further south (Fig 3), will also be referred since it is the latest Medieval site with ^14^C dates and enough grains to allow a biometric characterization.

In total, over 100,000 remains of rye were collected (Tables 2 and S2). The majority of the remains comprised grains, whereas chaff was usually scarce. The macroremains were always preserved by charring, except four waterlogged internodes from Ponte do Burgo (PDB) [71]. The Roman/Late Antique and Medieval contexts display higher amounts of rye, but these are overrepresented by data from CSJAMD (96,4% of all Roman/Late Antique remains) and SB results (almost all Medieval remains). Still, a significant quantity of remains was recovered from Iron Age/Early Roman deposits. Cereals were often dominant in the fruits/seeds assemblages. The frequency of rye in the assemblages and archaeological contexts varied significantly, being either dominant or secondary (S2 Table), and scarcely or widely represented in the deposits (Table 2).

**Table 2:** Rye record per site and period. For more details, see S2 Table.

		Rye					
Period	Sites	Grains	Chaff	Total Fruits/Seeds	Rye %	Rye ubiquity per S.U.	%
Iron Age/Early Roman	CPS	2,555	513	5,591	54.9	1/1	100
	CRT	624	7	91,812	0.7	5/10	50
	CDCR	6	6	2,183	0.5	6/51	11.8
	CRUI	13	0	≈170	≈7.6	No information	N/A
	TONG	4	0	1,860	0.2	3/20	15
Roman/Late Antiquity	CSJAMD	49,186	0	145,072	33.9	9/9	100
	MOZ	552	1,171	17,307	10	19/31	61.3
	CDCR	3	43	333	13.8	9/11	81.8
	GUIF	28	14	285	14.7	6/9	66.7
	CRES	11	0	35	31.4	5/10	50
	TONG	11	0	245	4.5	6/8	75
	QCREST	6	0	52	11.5	3/12	25
	AST	1	0	36	2.8	1/1	100
	CDG	1	0	1	100	1/1	100
Medieval	SB	48,237	1,712	154,846	32.3	23/24	95.8
	ARF	5	0	269	1.9	3/5	60
	PDB	0	4	1,014	0.4	1/2	50
	QCREST	1	0	40	2.5	1/9	11.1

### Radiocarbon dating, Bayesian and Kernel Density analysis

**Fig 4:**
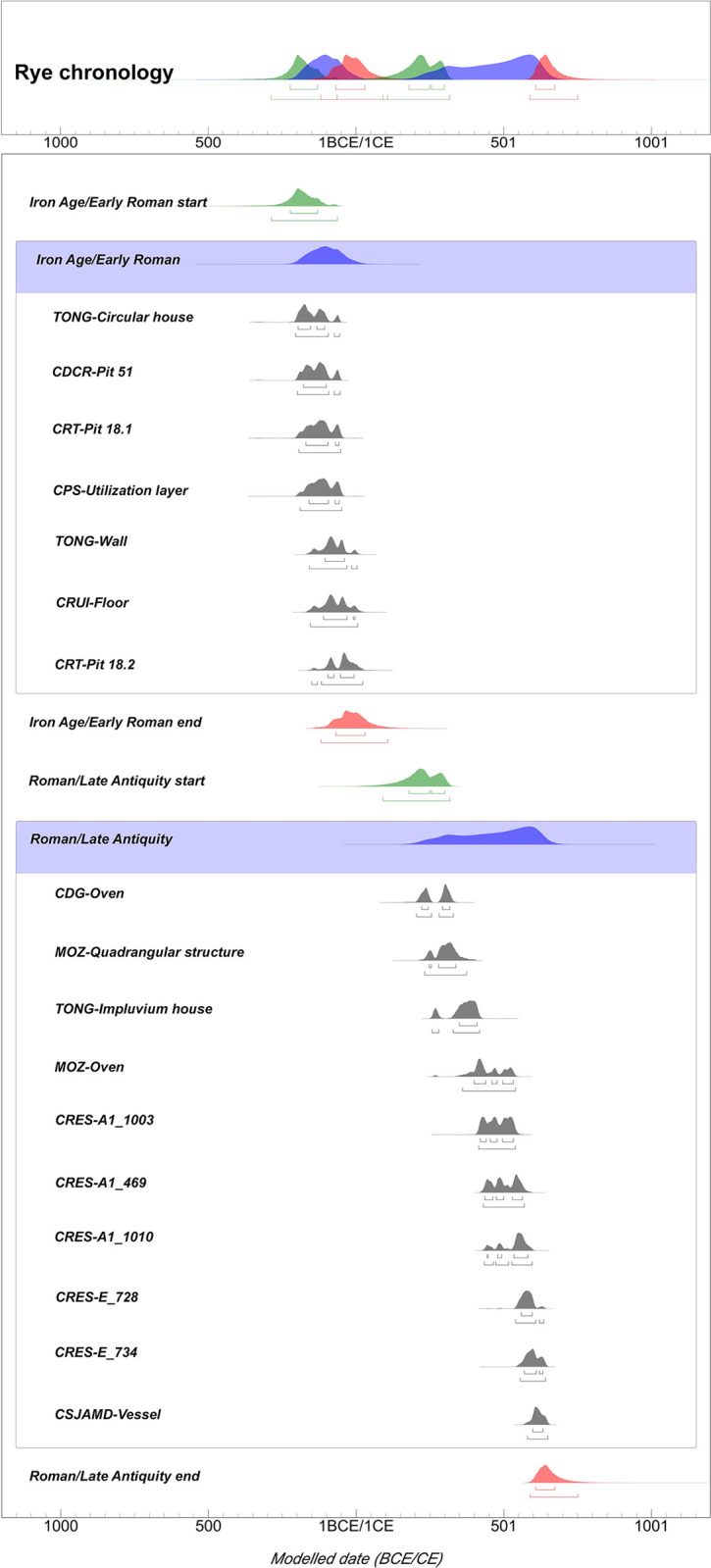
OxCal plots of the Bayesian and KDE analysis. Above: Stacked plot with start (green) and end (red) boundaries as well as the KDE_Plots of each group (blue). Below: Multiple plot including the two sequential phases and the respective boundaries as well as the modelled data (dark grey) of each radiocarbon date. Model carried out through OxCal 4.4.4 software [87], IntCal20 calibration curve [97]. All modelled ages were rounded to the nearest five years, following Bayliss and Marshall [98].

Eighteen radiocarbon dates were obtained on grains of rye from 10 sites (Table 3). A combination of Bayesian and Kernel Density analysis was conducted (Fig 4), with dates added to an individual sequence with two phases (Iron Age/Early Roman and Roman/Late Antiquity). SB was excluded from this analysis because it was the only site with a Medieval ^14^C date. The multiphase model (sequential) revealed positive results (see S3 Table), showing strong agreement indices (*Amodel = 86*.*7%*, *Aoverall = 82*.*2%*), well above reference values [96].

**Table 3:** Rye ^14^C dates. Calibration OxCal 4.4.4 software [87], IntCal20 calibration curve [97]. The calibrated ages were rounded to the nearest five (^14^C dates with uncertainties below 25) and 10 (^14^C dates with uncertainties equal to or above 25) years, following Bayliss and Marshall [98].

Abbreviation	Context	Period	Lab. Ref.	14C age (yr BP)	Calibrated age BCE-CE (68.3%)	Calibrated age BCE-CE (95.4%)	References
CDCR	Pit 51	Iron Age	D-AMS 044739	2139 ± 20	340–115 BCE	350–55 BCE	[99]
CRT	Pit 18.1	Iron Age	D-AMS 016318	2132 ± 31	340–100 BCE	350–40 BCE	[74]
	Pit 18.2	Late Iron Age/Early Roman	D-AMS 011304	2027 ± 25	50 BCE–20 CE	100 BCE–70 CE	
CPS	Utilization layer	Iron Age	Beta-596126	2120 ± 30	180–50 BCE	350–40 BCE	this paper
TONG	Round hut	Iron Age	D-AMS 047814	2158 ± 21	350–160 BCE	355–105 BCE	this paper
	Wall	Late Iron Age/Early Roman	D-AMS 042655	2062 ± 22	105 BCE–5 CE	155 BCE–10 CE	this paper
	*Impluvium* house	Late Roman	D-AMS 009829	1692 ± 27	260–410 CE	250–420 CE	[101]
CRUI	Floor	Late Iron Age/Early Roman	Beta-349645	2050 ± 30	100 BCE–10 CE	160 BCE–30 CE	this paper
CDG	Oven	Roman	D-AMS 044740	1819 ± 23	210–310 CE	130–325 CE	this paper
MOZ	Quad. structure	Late Roman	Beta-309441	1770 ± 30	240–340 CE	220–380 CE	[70]
	Oven	Late Antiquity	Beta-349646	1640 ± 30	400–540 CE	360–540 CE	[126, 127]
CRES	A1-1003	Late Antiquity	D-MAS 9672	1607 ± 26	420–540 CE	410–540 CE	this paper
	A1-469		D-MAS 8868	1557 ± 24	435–565 CE	430–570 CE	
	A1-1010		D-MAS 8871	1538 ± 24	440–580 CE	435–600 CE	
	E-728		D-MAS 8877	1501 ± 24	555–600 CE	540–640 CE	
	E-734		D-MAS 8879	1471 ± 28	570–640 CE	560–650 CE	
CSJAMD	Vessel 1	Late Antiquity	D-AMS 042654	1435 ± 24	605–645 CE	585–655 CE	[128]
SB	Wall filling	Medieval	Beta-46513	1170 ± 30	770–950 CE	770–980 CE	[141]

Seven ^14^C dates provided calibrated ranges between the Iron Age (4^th^ century BCE) and the Early Roman period (1^st^ century BCE/1^st^ century CE). The oldest dates from TONG (Round hut), CDCR, CRT (Pit 18.1), and CPS showed similar calibrated results with long intervals (355–40 BCE at 95.4% probability). There is only a difference of around half a century concerning the date from TONG (Round hut), which shows a shorter interval (355–105 BCE). The other ^14^C dates from TONG (Wall), CRUIT, and CRT (Pit 18.2) fell within a more recent period, between the 2^nd^–1^st^ centuries BCE and the 1^st^ century CE.

The modelling data point to a shorter time frame, suggesting rye appeared in Northwest Iberia between *290–60 BCE* (*95*.*4%*) with the *68*.*3%* range showing a date range between *225–130 BCE* (S3 Table). Following the results, rye should have persisted in the region until *120 BCE–110 CE* (95.4%), while the *68*.*3%* confidence interval mentions a date range between *70 BCE–35 CE*. The probability distribution of the Iron Age/Early Roman KDE Plot reveals a higher concentration of dates starting around the late 3^rd^ century BCE and reaching its peak approximately at the end of the 2^nd^ century BCE, followed by a small drop and a progressive fall from around the mid-1^st^ century BCE to the early 1^st^ century CE (Fig 4).

Ten ^14^C dates provided Roman and Late Antique chronologies. The calibrated results indicate a Roman chronology for three dates from CDG, MOZ (Quad. structure), and TONG (*Impluvium* house), which span from the 2^nd^ century CE to the early 5^th^ century (130–420 CE). Despite the broad time span, the majority of the data point to chronologies between the 3^rd^ century and the early 5^th^ century CE. Only the ^14^C date from CDG shows a 95.4% range (130–325 CE) outside this period. The other indicators, including from CDG suggest a chronology posterior to the 2^nd^ century CE. The remaining seven Late Antique dates fall between the second half of the 4^th^ century and the 7^th^ century CE (360–655 CE) with almost all probabilities pointing to chronologies between the 5^th^ century and the 7^th^ century CE.

The Bayesian model displays a slight overlap between the two phases, namely between the *95*.*4%* confidence intervals for the end of the Iron Age/Early Roman (*120 BCE–110 CE*) and start of the Roman/Late Antiquity (*90–320 CE*). Nonetheless, the rest of the modelled data point to a separate moment. The *68*.*3%* range suggests that rye appears again between *180–300 CE*. The Interval function in OxCal displays gaps between the two phases from *55* to *380* years (*95*.*4%*), and from *160* to *320* years (*68*.*3%*) (S3 Table). These results strongly support a gap between the two phases, at least of around two centuries. The distribution of the Roman/Late Antique KDE_Plot also strengthens this idea, showing the first concentration increasing only in the late 2^nd^ century with a peak around the early 4^th^ century CE. It shows a second and higher peak at ca. 600 CE. However, this last result is highly determined by the several dates obtained in a single site, namely CRES, where rye seems to have been present in consecutive settlement phases.

### Biometric approach

Measurements varied considerably among the 457 analysed grains: length (3.66–6.99 mm); width (1.43–3.19 mm); and thickness (1.35–3.15 mm). There are differences between distinct chronologies, but there is no linear chronological pattern (Table 4).

**Table 4:** General results of the biometric study. Minimum (Min.), maximum (Max.), mean, and standard deviation (SD) values of each measurement point (length, width, thickness).

Sites	Length (mm)				Width (mm)				Thickness (mm)			
	Min.	Max.	Mean	SD	Min.	Max.	Mean	SD	Min.	Max.	Mean	SD
CRT	5.07	6.54	5.75	0.39	1.62	2.81	2.14	0.24	1.54	2.78	2.08	0.24
CPS	4.34	6.32	5.26	0.41	1.51	2.2	1.86	0.16	1.42	2.17	1.79	0.15
MOZ	3.66	6.88	5.21	0.63	1.43	2.86	2.2	0.32	1.35	2.85	2.08	0.3
CSJAMD	4.72	6.99	5.63	0.46	1.63	2.57	2.08	0.17	1.63	2.52	2.03	0.16
SB	4.53	6.59	5.64	0.48	2.07	3.19	2.49	0.24	1.93	3.15	2.41	0.26

In terms of length, grains from MOZ and CPS are shorter (means of 5.21 mm and 5.26 mm, respectively) (Table 4). Those of MOZ show a large variation, with values ranging from 3.66 mm to 6.88 mm (SD of 0.63). Still, the majority show values between 4 mm and 6 mm. Results in CPS are more homogeneous (SD of 0.41), with grain length frequently between 5 mm and 6 mm. CSJAMD, SB, and CRT revealed identical results. Although grains in CRT grains are, on average, slightly longer (5.75 mm), measurements were only conducted on 57 units.

In every site, grains display higher width than thickness, but differences are small. CPS grains are narrower and thinner (means of 1.86 mm and 1.79 mm, respectively) while the remaining assemblages show average values above 2 mm regarding both width and thickness. Among these, CSJAMD, MOZ, and CRT showed similar measurements. Grains from CSJAMD are narrower (mean of 2.08 mm) than CRT (2.14 mm) and MOZ (2.2 mm) grains, but differences are negligible, and limitations may be pointed again for CRT (limited number of grains) and MOZ (high standard deviation). Measurements of thickness provided similar results. CSJAMD grains are slightly thinner (2.03 mm), but those of MOZ and CRT grains are not very different (2.08 mm). At SB, the most recent site, grains are plumper. This site provided the highest mean values both in terms of width (2.49 mm) and thickness (2.41 mm), and in each case almost all grains had more than 2 mm, some even surpassed the 3 mm.

**Fig 5:**
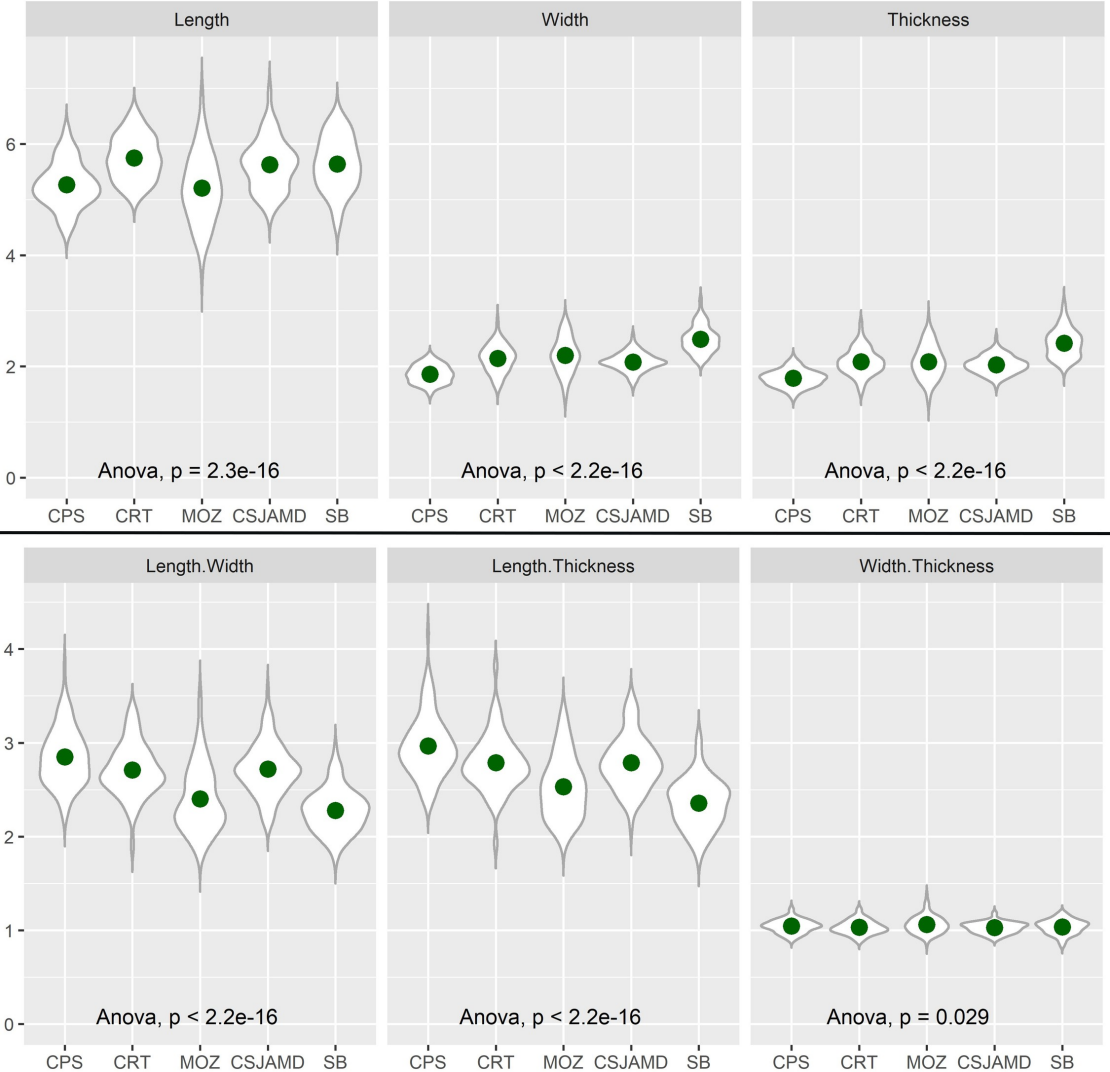
Graphical representation of the measurements and some of the ratios obtained (see full details in S1 Fig). Sites are arranged chronologically from left (earlier) to right (later).

Most ratios did not reveal clear insights (Figs 5 and S1). Two contrasting cases were observed, namely CPS and SB. CPS shows high L/W and L/T ratios, as well as low W/L*100, whereas SB presents the opposite. These results suggest that CPS grains had a more elongated and slender form than SB grains which were more compact and fatter. MOZ provided results closer to those from SB, showing similar L/W and L/T ratios. CRT and CSJAMD grains revealed almost indistinguishable ratios, and a more proximity to CPS results.

## Discussion

### Rye in the Iron Age and Early Roman periods: Crop or weed?

Macroremains of rye were found in five Iron Age/Early Roman sites in the Northwest in multiple archaeological contexts and integrated in archaeobotanical assemblages with different characteristics (Tables 2 and 3 and S2). The dated Iron Age/Early Roman grains correspond to the oldest secure evidence of rye in the Iberian Peninsula. The Bayesian modelling suggests its introduction occurred between the 3^rd^ century and the first half of the 1^st^ century BCE (95.4%), most probably between the 3^rd^ and 2^nd^ centuries BCE (68.3%). The strong distribution of the Iron Age/Early Roman KDE_Plot within this last period of time also supports this chronology.

Rye is usually scarce in the study area and is only dominant in CPS. This site shows the largest concentration of chaff and grains of rye, whereas hulled wheats are secondary in number. However, the rye grains are mostly compressed inwards, a characteristic that could be associated with the charring of the still green kernels. This suggests that rye, although the most numerous species in the samples, may have been a secondary cereal that was gathered as part of an already ripe hulled wheat harvest.

A different scenario was observed at CRT. Abundant rye grains were found together with few chaff remains, including grains within the spikelets. Those were collected from inside storage pits where spelt was largely dominant [74]. In the remaining sites, namely CDCR, CRUI, and TONG, only a few remains of rye were collected and other cereals were more common, such as millets, hulled or naked wheats [99–101].

Considerations about the domestic/weedy character of rye during the Iron Age and Early Roman times are problematic. Despite the robust chronological framework, the characterization of the archaeobotanical assemblages and the biometric data did not provide straightforward conclusions about this issue. On the other hand, it does not support the presence of other morphologically similar cereals in the assemblages here studied, such as other rye species or *Dasypyrum villosum*. In fact, some data suggest rye might have been considered a weed at this time.

If abundance and predominance are the main criteria to identify problematic taxa as crops, the remains of CPS would promptly be considered as cultivated. However, the interpretation of the archaeological context where the remains were found must not be disregarded. In this case, plant remains were found in a small concentration, in a context whose interpretation was rather difficult. It was probably the result of a short-term event and the representativeness of this assemblage in the occupation of the site was impossible to assess. In addition, despite grains of rye are easily freed in the early stages of processing, they were found in association with large amounts of chaff and some were still within the spikelets. While such easy and early dehusking often leads to an underrepresentation of chaff from free-threshing cereals, like rye, in the archaeobotanical record (e.g. [102,103]), the presence of unprocessed rye could support its weedy character, being rye eventually tolerated and processed with other cereals, such as hulled wheats. The record of naked wheat grains at CPS was restricted but the absence of any chaff also seems to support this interpretation. Besides, biometric data revealed that grains from CPS are, on average, thinner and less thick than most. Considering all this, we cannot reject the possibility that grains were immature when charred, as aforementioned. In the future, experimental work must be conducted in order to test this hypothesis. Nonetheless, the amount of remains recovered in CPS is noteworthy.

On the other hand, at CRT spelt was largely dominant. Rye, comprising around 1% of the assemblage, may have been one of its weeds. Such kind of interpretations were proposed for analogous assemblages found in Iron Age sites from central Europe (e.g. [104,105]). However, grains from CRT showed larger dimensions than those from CPS, being closer to those from the Late Antiquity (MOZ, and CSJAMD) regarding width and thickness. The different ^14^C dates (Pits 18.1 and 18.2) showed that rye was present in two or more moments at CRT, perhaps in a recurrent way. It is possible that the frequent seeding of rye, deliberate or not, together with other cereals, led to an increase in grain size.

CRT is not a single case in the region. Rye has been consistently found in association with hulled wheats, and mainly with spelt [99–101]. It is true that spelt was one of the main crops in the Iron Age. It was found in many sites together with other domesticated cereals, such as millets, barley and free-threshing wheat. Therefore, this association alone cannot be used to support the weedy character of Iron Age rye. However, when spelt almost disappears from the archaeobotanical record in the first centuries CE, rye also ceases to be found, only to appear again as a crop in a later moment of the Roman Empire (see below). This suggests rye may have been a weed in the fields of spelt in the Iron Age. Despite the number of sites from the 1^st^–2^nd^ centuries CE that have been studied is still low [62,79,106], this hypothesis seems plausible.

Either way, the identification of rye in several archaeological sites and different occupation phases suggest rye was often part (consciously or not) of the agricultural fields from the Iron Age to the Early Roman Empire, in some areas of Northwest Iberia. The similar dimensions of grains from CRT and Late Antique deposits indicate they did not develop much between the two periods. The large grains of CRT may eventually be the result of the undeliberate selection of grain and its frequent seeding, but more extensive work, extended to other regions, is needed to shed some light on this matter.

Interestingly, Iron Age/Early Roman sites where macroremains of rye have been found so far are located in a small area, mostly in the Sousa and Tâmega rivers basins (subsidiaries of the Douro river) as their vicinities. In this early chronology, rye is absent from the westernmost sites, near the Atlantic, where it is found only in Late Antique sites (Figs 2 and 3). This is hardly the result of any investigation bias, considering the amount of sites studied in the region. Only future research will confirm or dismiss this pattern. Rye is absent elsewhere in Iberia during this period, although it is recorded in the French Pyrennes, namely at La Chance (Villefranche-de-Conflent), in a Neolithic context [107] and at Llo-Lo Lladre (Llo) in Late Bronze Age deposits [108]. In both cases, few grains were recovered and likely correspond to weedy or perhaps wild remains.

Although pollen data suggest the presence of *Secale* taxa long before the chronologies here obtained, caution is necessary in the interpretation of those remains, particularly concerning the earliest findings. Mesolithic, and Neolithic findings were rare and discontinuous in the pollen curves of the Iberian Peninsula (see Rye in Iberia–The pollen evidence). Pollen evidence dating to the Mesolithic is well representative of how problematic this evidence is. It was noteworthy the identification of a wider distribution of *Secale* taxa in Bronze and Iron Age deposits, but only through pollen data is not possible an association with agricultural practices, especially when remains are present in reduced amounts. Besides, eventual contaminations and misidentifications may be also considered (e.g. [17,37,109,110]). Moreover, the native distribution of mountain rye (*Secale montanum*) includes the Mediterranean basin, being mainly found in mountains of Central and Southern Iberia as well as in the Eastern Pyrenees, although it is currently rare (e.g. [4,5,111–117]). The record of one macroremain (grain) of mountain rye at Cova de les Cendres (Alicante, Eastern Iberia) indicates the presence of this species in the Late Gravettian [118]. Thus, and despite the actual information concerning the natural range of mountain rye does not cover all Iberian regions where earlier pollen remains were found (see Rye in Iberia–The pollen evidence), an eventual association with this wild rye is admissible. Both mountain and domesticated rye pollen overlap significantly in terms of size, and the attribution of *Secale* taxa to wild rye species, and even other wild Poaceae, was already admitted in other studies [37,109,119].

Despite the pollen record, the presence of macroremains of rye in the Iron Age/Early Roman period in Northwest Iberia is difficult to explain. The late 2^nd^ century BCE is a period of changes in this geographical area, characterized by population concentrations in large fortified settlements, the *oppida*. It is also the time of the first Roman incursions. These started in 138–137 BCE and throughout the 1^st^ century BCE the region went under Roman influence (e.g. [120–122]), even though indigenous communities likely maintained their way of living. Small and temporary military sites were found (e.g. [123,124]) but the three administrative capitals in Northwest Iberia (*Bracara Augusta*, *Asturica Augusta* and *Lucus Augusti*) were founded by the end of the 1^st^ century BCE, after which the Roman control of the region became more effective. The introduction of rye is close to the early stages of this change but it is unclear how it relates to it. If it was a crop, it could have been introduced by Roman influence, but rye was not a staple anywhere in the Empire at the time. If it was a weed in the fields of spelt, as suspected, is strange why it was not found in association with spelt in earlier periods. Another possible scenario is the arrival of new spelt varieties and their corresponding weeds including rye (e.g. via trade contacts or as a result of population displacement), but it is unclear why rye has been only found in a small area and not in wider regions of the Iberian Peninsula despite the efforts in sampling for archaeobotanical analysis in recent decades [125]. A better understanding of this phenomenon demands a more thorough review of old collections and, mostly, new archaeobotanical work. Since rye is usually rare in the assemblages, ambitious sampling strategies are needed to increase the chances of finding remains.

### The (re)introduction of rye

After a gap of around two centuries, rye is again found in Roman times (Tables 2 and 3 and S2). Due to the large date ranges of several of the ^14^C dates as well as some of the modelling data (see above), rye most likely reappeared in Northwest Iberia between the late 2^nd^ century CE and the turn to the 4^th^ century CE (68.3%). This last chronology is close to that from other sites in Northeast Iberia [53,54], suggesting rye was cultivated in more areas of the peninsula during a later period of the Roman Empire. Late Antique deposits in Northwest Iberia, as well as several ^14^C dates, suggest rye continued to expand in the 5^th^–7^th^ centuries CE (Tables 2 and 3 and S2 and S3).

Overall, rye was found in nine Northwestern sites with Roman and Late Antique occupations. Still, only two sites provided significant amounts of remains. At MOZ rye was recovered in features from two consecutive phases, the 3^rd^–4^th^ centuries CE and the 4^th^–6^th^ centuries CE, including a storage structure, and an oven and a debris area. In the later period it was even dominant [70,126,127]. At CSJAMD, rye was the dominant crop in a storage context destroyed by fire in the 6^th^–7^th^ centuries CE, where almost 50,000 grains were found in above-ground stone storage facilities and their surroundings. Several of them were inside ceramic vessels [128]. It is clear rye was then a crop. Nonetheless, biometry data do not show substantial differences between grains from MOZ and CSJAMD, or between these and those from CRT dating to the Iron Age/Early Roman period. In other Northwestern Roman and Late Antique contexts rye is rare (CDG, CDCR, TONG, CRES, GUIF, QCREST, AST), and, overall, these provided small fruits/seeds assemblages [99,101,129–131]. It was also recorded in several sites from Northeast Iberia, and also in the Visigothic village of Gózquez in Central Spain, dating to the 6^th^–8^th^ centuries CE [54,55,58,60,64,66]. Despite the caution that pollen data demand (see above), the increasing presence of *Secale* taxa after the Roman period in Iberia seems to strengthen the idea that rye cultivation expanded during Late Antiquity.

It has been suggested that the spread of rye in some European regions during Late Antiquity was due to environmental reasons (e.g. [132]). After ca. three centuries of relatively stable and favorable (i.e. warm and humid) conditions, a period of climatic instability, generally colder and drier, began in the 3^rd^ century CE. Despite climatic amelioration in the 4^th^ century, as wetter and warmer conditions prevailed, in the 5^th^ and 6^th^ centuries there was growing climatic instability, with warming and cooling periods being recorded [133]. The suitability of rye for these conditions is undisputable, but environmental factors alone are insufficient to explain the expansion of rye cultivation [134].

Germanic influence throughout the imperial areas increased significantly from the 3^rd^ century onwards, culminating in 411 with a treaty in which Rome delivered control of Northwest Iberia to the Suebi, hence starting the Suebian Kingdom with its capital at Braga [135]. Considering that rye was a relevant crop in Central Europe since earlier periods, it is possible that these increasing contacts and migration led to the dissemination of this crop in the westernmost areas of the Roman Empire. Other structural issues might have helped. Throughout Late Antiquity, as subsistence farming expanded in the context of some ruralisation, rye was a relevant choice as it increased the resilience of agrosystems [134]. Furthermore, after the great agricultural development and subsequent soil degradation of the Iron Age and Roman period in Northwest Iberia, rye would allow the use of less favourable soils [79].

Still, as Squatriti [134] refers, rye was not the only crop that expanded in the Late Antiquity. The hypothetic reintroduction of rye could be comprehended as part of the larger environmental, economic, cultural and population dynamics that characterize the end of the Western Roman Empire and the beginning of the Medieval period. In Northwest Iberia, as in other regions, sweet chestnut (*Castanea sativa*) became more frequent in archaeobotanical assemblages dating to this period (e.g. [101,106,126,127,131]), suggesting this tree, although autochthonous in the region, was cultivated at the time [136]. Rye could have replaced the role fulfilled by hulled wheats, which are more resistant to pests and adverse weather conditions than naked wheats, and more adaptable to a winter growing season. The higher green fodder productivity of rye, in addition to their better growth on poor soils, could have meant an advantage over emmer and spelt in the cropping cycles.

### The spread of rye in Medieval times

**Fig 6:**
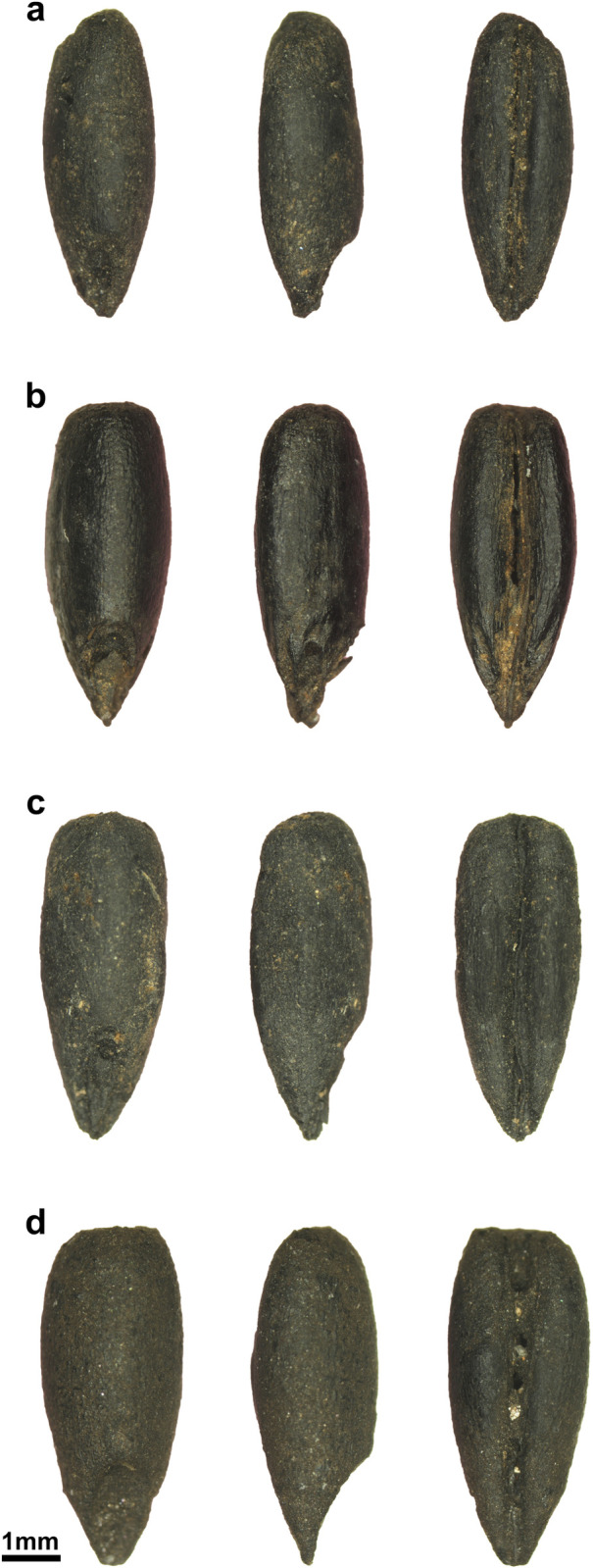
Rye grains (dorsal, lateral, and ventral views) from all analysed periods. Legend: a) CRT (Iron Age/Early Roman), b) MOZ (Late Antiquity), c) CSJAMD (Late Antiquity); d) SB (Medieval). Scale 1mm.

The key role of rye during Medieval times is supported by archaeobotanical evidence and written sources (e.g. [1,76,77,137–140], showing rye disseminated across Iberian regions with different cultural backgrounds (S1 and S2 Tables). Nearly all medieval rye macroremains from this study came from SB, in Central Portugal. Here, a large and diverse fruits/seeds assemblage was recovered, rye and oat being the main crops [141]. The biometric results revealed that SB rye grains were broader and thicker than the other older measured grains (Fig 6). It is possible thus that rye grain increased in size during Medieval times, but additional grain-rich assemblages are needed to confirm this hypothesis.

At SB, other cereals were abundant, such as hulled barley or broomcorn millet, while foxtail millet and naked wheat were less represented. In other sites (Tables 2 and S2), fewer fruits/seeds were collected, and rye remains were always rare [71–73,129]. Still, rye was found in other sites in Western Iberia (S1 Table), south from the Douro river, such as São Gens [142], Convento de São Francisco de Santarém [143] and Quinta da Torrinha [144].

Rye was commonly mentioned in Medieval written documentation from Northwest Iberia, suggesting its cultivation was widespread, particularly, but not exclusively in areas with harsh climates and poor agricultural soils (e.g. [76,77]). Such sources became frequent from the 13^th^ centuries onwards but, as noticed before, archaeobotanical data from our study area suggest such dissemination occurred much earlier. The pollen record also seems to suggest a more important role of rye in agricultural strategies during this period, being *Secale* taxa present in numerous sites in the Iberian Peninsula, although frequently in small amounts (see above Rye in Iberia–The pollen evidence).

## Final remarks

This research addressed the introduction and dissemination of rye through archaeobotanical, biometric, and radiocarbon analyses. As a result, it is suggested that rye first appeared in Northwest Iberia, during the Iron Age, probably as a weed. The ^14^C and modelling results suggest its introduction occurred between the 3^rd^ century and the first half of the 1^st^ century, probably between the 3^rd^ century and the 2^nd^ century BCE. Rye must have prevailed in the agricultural fields of the region until the turn of the era, likely associated with spelt. Although rye is dominant in one site and biometric data do not suggest grains increased in size from the Iron Age to the Late Antiquity, it is more frequently a secondary, sometimes rare cereal in the earlier phase. The development of morphometric analyses may clarify this matter in the future.

So far, the chronology of rye´s arrival is coincident with changes in the local settlement and the first Roman incursions, but the short geographic area of the earlier findings, the absence of rye in the assemblages with spelt outside this small area, and doubts regarding how the introduction of rye is related to those dynamics make the interpretation rather difficult. Besides, we are missing macrobotanical data from the rest of the Iberian Peninsula. The nearest clear pre-Roman macroremains of rye were found in the French Pyrenees and in small amounts. The earlier record of *Secale* taxa in the pollen curves is conditioned by several factors, not allowing secure considerations. Thus, more archaeobotanical studies are needed, both in Northwest Iberia and in other Iberian regions, to better understand the context of rye introduction and expansion. The same could be mentioned for other European areas. Isolated findings and small concentrations of rye likely need a reassessment and a wider integration to understand its introduction, dissemination, and transformation from weed to crop within the continent.

After a gap of about two centuries, rye was introduced as a crop at a later time during the Roman Empire in Northwest Iberia and in other Iberian regions. Throughout Late Antiquity, rye was increasingly recurrent and abundant in the archaeobotanical record of Iberia, as well as of other European areas [1,132,134,138]. Its dissemination may be due to a combination of cultural and environmental factors. Rye´s adaptability to harsh environments and poor soils was surely a relevant factor, but the population and political dynamics of the epoch were likely as crucial. Increasing migration from Central Europe, where rye was cultivated before, and the dissemination of small and self-sufficient rural settlements probably helped giving rye a further weight in the agricultural strategies of less favourable areas. The full spread of rye was already clear in the Medieval period. It was identified in archaeobotanical assemblages from nearly all Iberian regions, either in Islamic or Christian archaeological contexts.

## Supporting information

S1 Fig Figures obtained through the statistical analyses carried out in R (biometric approach). (PDF)

S1 Table Sites with macroremains of rye in Iberian Peninsula until the Late Medieval period (15^th^ century CE), and the amount of both grains and chaff recovered in each one of them. (XLSX)

S2 Table Representativeness of rye in the archaeobotanical record of the 15 sites addressed in this approach. Analysis per period, including the identification of the predominant cereal and other cereals as well.
(XLSX)

S3 Table Table with the results of the KDE and Bayesian analysis, including only modelled data (adapted from the OxCal software (version 4.4.4.) [87] and applying IntCal20 calibration curve [97]). The Medieval ^14^C date from SB was not included. All modelled ages were rounded to the nearest five years, following Bayliss and Marshall [98].
(XLSX)

S1 Text Description of the 10 archaeological sites with radiocarbon dates over grains of rye. (DOCX)

S2 Text OxCal code (CQL2) used to create the multiphase model. (DOCX)
