# Supplementary material for: The introduction and spread of rye (Secale cereale) in the Iberian Peninsula
Source: PLoS One. 2023 May 10;18(5):e0284222. doi: 10.1371/journal.pone.0284222 (PMC10171662; doi:10.1371/journal.pone.0284222)
Supplement: S1 Fig — (PDF) [file pone.0284222.s001.pdf]

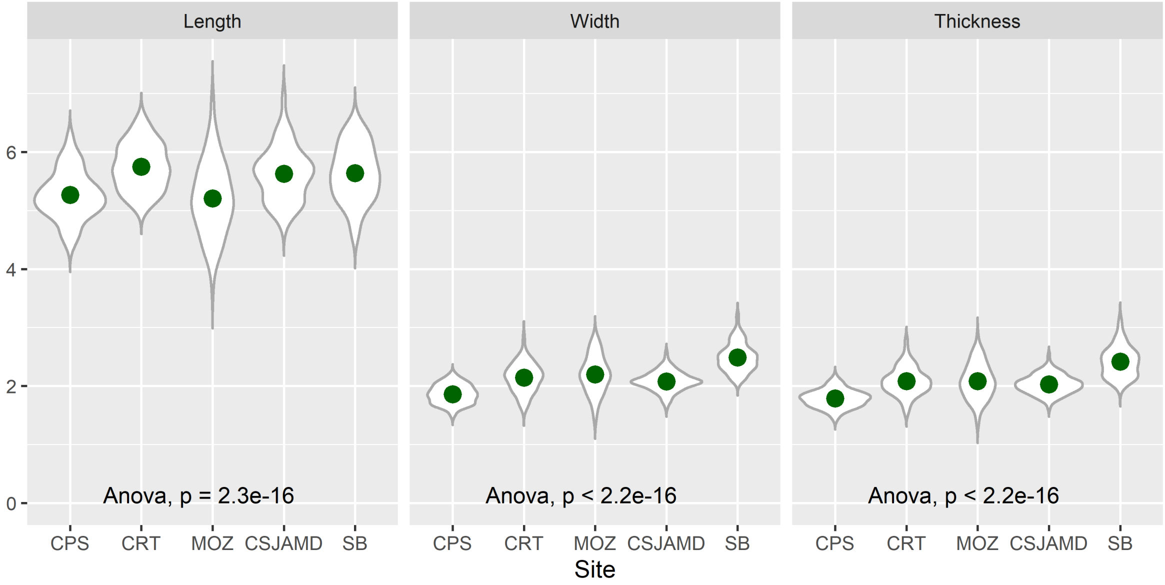
Length
Width
Thickness
6
4
2
0
Anova, p = 2.3e-16
Anova, p < 2.2e-16
Anova, p < 2.2e-16
CPS
CRT
MOZ
CSJAMD
SB
Site

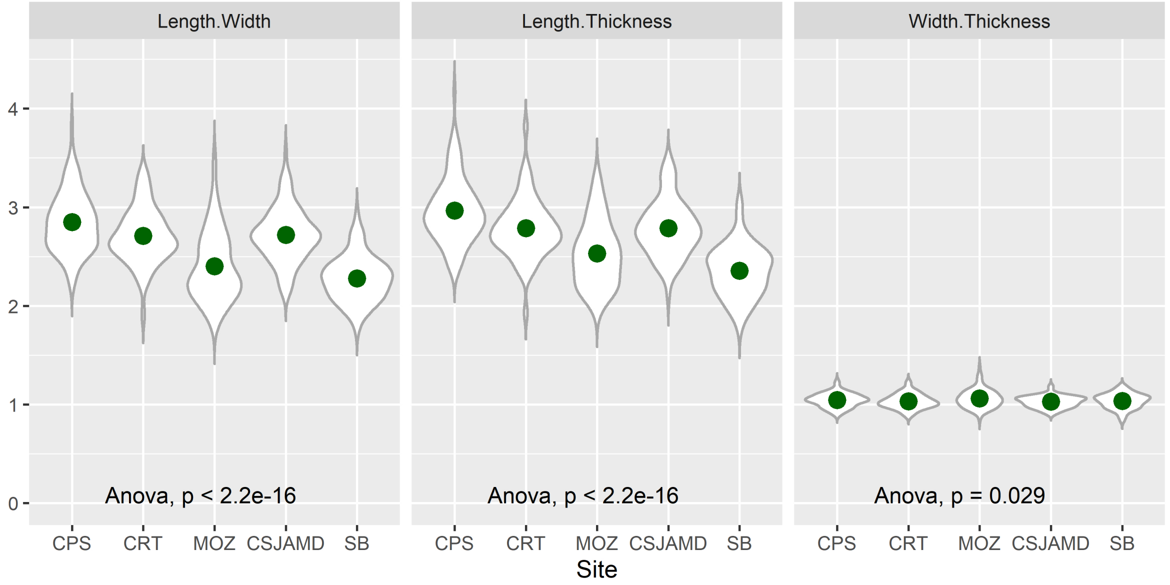

Length.Width
Length.Thickness
Width.Thickness
Anova, p < 2.2e-16
Anova, p < 2.2e-16
Anova, p = 0.029
0
1
2
3
4
CPS
CRT
MOZ
CSJAMD
SB
Site

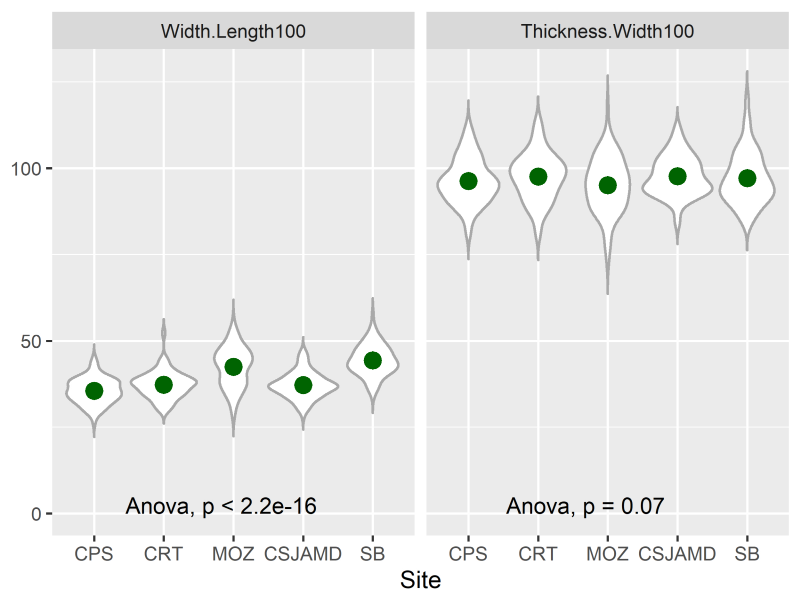

Width.Length100
Thickness.Width100
100
50
0
Anova, p < 2.2e-16
Anova, p = 0.07
CPS
CRT
MOZ
CSJAMD
SB
Site

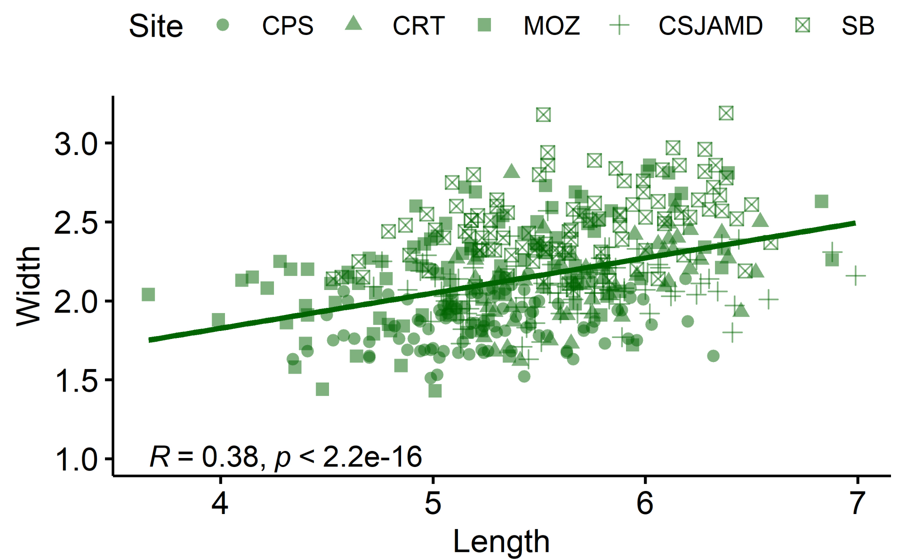

Site
CPS
CRT
MOZ
CSJAMD
SB
Width
3.0
2.5
2.0
1.5
1.0
R = 0.38, p < 2.2e-16
4
5
6
7
Length

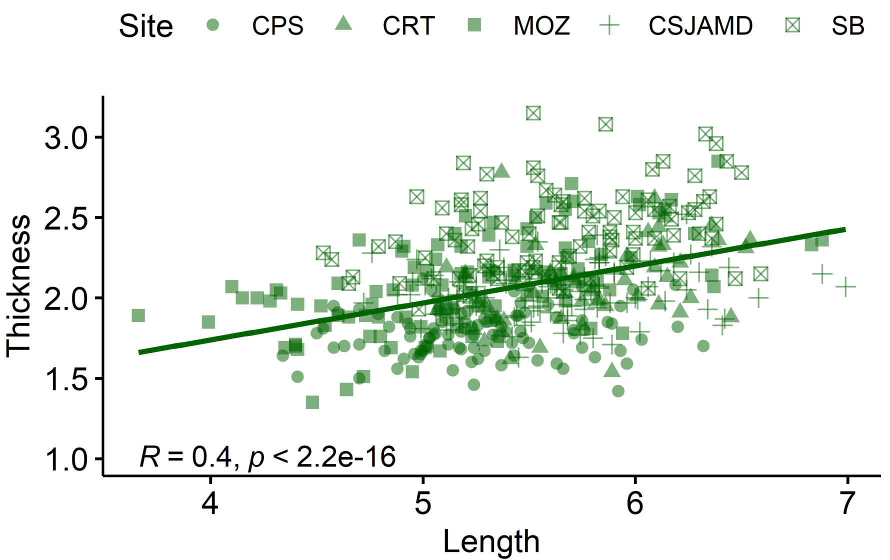

Site
CPS
CRT
MOZ
CSJAMD
SB
Thickness
3.0
2.5
2.0
1.5
1.0
4
5
6
7
Length
R = 0.4, p < 2.2e-16

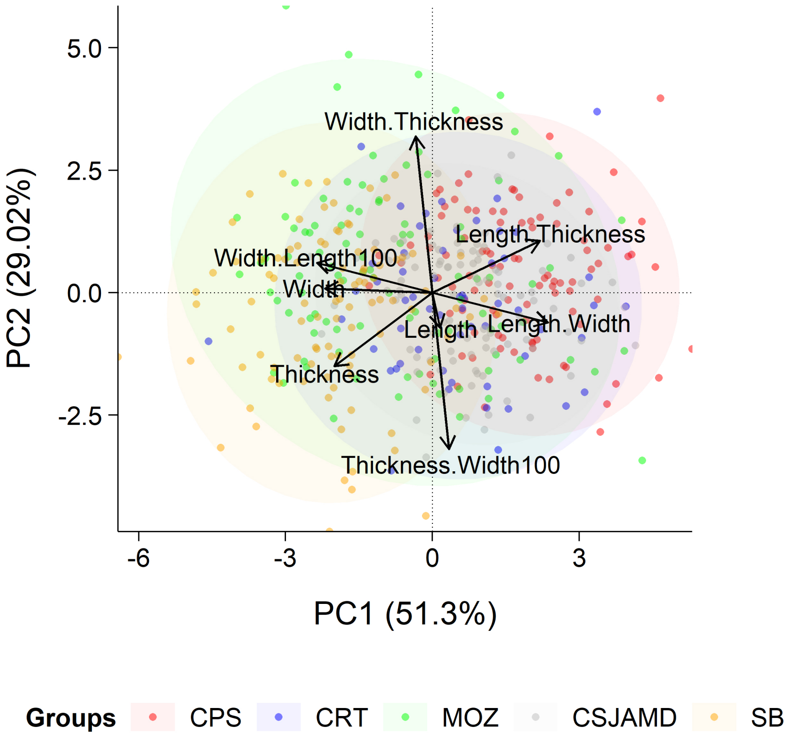

Width.Thickness
Length.Thickness
Width.Length100
Width
Length
Length.Width
Thickness
Thickness.Width100
5.0
2.5
0.0
-2.5
-6
-3
0
3
PC2 (29.02%)
PC1 (51.3%)
Groups
CPS
CRT
MOZ
CSJAMD
SB
