# Supplementary material for: The introduction and spread of rye (Secale cereale) in the Iberian Peninsula
Source: PLoS One. 2023 May 10;18(5):e0284222. doi: 10.1371/journal.pone.0284222 (PMC10171662; doi:10.1371/journal.pone.0284222)
Supplement: S1 Text — (DOCX) [file pone.0284222.s005.docx]

**Supporting Information (S3 Text)**

**Description of the 10 archaeological sites with radiocarbon dates over grains of rye.**

**1- “**Casa Romana” (Roman house) of the Castro de São Domingos (CDCR): located in the lower slopes of an Iron Age hillfort, at around 314 m a.s.l., CDCR integrates remains of several occupations covering a long period of time from, at least, the Iron Age to the Late Antiquity ([1-4]). The site is rather disturbed, but several structures have been identified, such as habitational areas, graves, postholes, and pits. Fruits/seeds analyses showed an assemblage dominated by broomcorn millet (*Panicum miliaceum*), a few grains and chaff of rye were recovered, so far, inside Iron Age negative structures and Late Antique graves ([5]). One grain from Pit 51 was dated, revealing an Iron Age chronology. The grains collected were not enough for the biometric analysis.

**2 -** Crastoeiro (CRT): placed on the slope of Monte Farinha, at 453 m a.s.l., CRT was an Iron Age hillfort with a subsequent but short Roman occupation ([6, 7]). In a central area, a large archaeobotanical assemblage was found in several storage pits. 624 grains of rye were found inside storage pits, most of them in Pit 18.2 ([8]). However, spelt (*Triticum spelta*) was predominant (55.1%), and rye represented just 1% of the fruits/seeds assemblage. Other cereals, namely broomcorn millet and hulled barley (*Hordeum vulgare*) were also more abundant than rye. Foxtail millet (*Setaria italica*) and naked wheat (*Triticum aestivum/durum*) were identified in smaller amounts. Two ^14^C dates obtained over grains of rye point to a period between the Iron Age and the Early Roman period. Due to preservation issues, only 57 grains were measured.

**3-** Castelo Pequeno de Santigoso (CPS): surrounded by several small streams and rivers, CPS was a large hillfort, implanted at 1,254 m a.s.l., with three enclosed areas defined by granitic walls. Excavations in 2019 have demonstrated occupations from the Iron Age and the Roman period ([9]). A very localized concentration of fruits/seeds was found in a utilization layer (s.u. 204), next to a delimitation wall, and a few metres below the hilltop. Rye is the predominant taxon as its chaff (n=513) and grains (n=2555) comprise 54.9% of the assemblage. It is followed at a distance by emmer (*Triticum dicoccum*) and spelt, while naked wheat is rare. One grain of rye was dated, revealing an Iron chronology. 100 grains were measured.

**4-** Cruito (CRUI): hillfort occupied between the 4^th^ century BCE and the 1^st^ century CE ([10]), located in the top of an elevation, at 516 m a.s.l., c. 4 km from the Douro river. Excavations carried out from 1984 to 1988, revealed several circular and rectangular houses, surrounded by two walls. In 1987, five samples were recovered and later studied by A.R. Pinto da Silva ([11]). A small number of rye grains was collected in an assemblage dominated by hulled and naked wheat. One grain was dated to the Late Iron Age/Early Roman period. The number of grains found did not reach the minimum number defined for the biometric study.

**5-** Freixo/Tongobriga (TONG): located at approximately 300 m a.s.l., close to the Tâmega river, TONG was an important Roman city in northwest Iberia, most likely a *civitas* under the administration of *Bracara Augusta* ([12]). Recent archaeological interventions have confirmed that TONG was inhabited since the Bronze Age and until the Middle Ages (e.g. [13-15]). A large number of ^14^C dates was obtained, several of them over rye grains. One of these was associated with a house occupied during Roman and Late Antique times, where foxtail millet was predominant. Other two were related to an Iron Age wall and a round hut that revealed archaeological evidence from the Iron Age and Late Roman times. In both contexts broomcorn millet was the main crop, and hulled wheats were found in significant amounts. A small number of grains was collected, insufficient for biometric analysis.

**6-** Cabeço da Grincha (CDG): small Roman farm, implanted at around 245 m a.s.l., a few metres from the Sabor river. The archaeological data suggest a short occupation, spanning from the second half of the 1^st^ century CE to the early 2^nd^ century CE ([16]). A single grain of rye, recovered in a destruction layer of an oven, was the only crop found in the site ([17]). The ^14^C date obtained revealed a long interval from the 2^nd^ century CE to the 4^th^ century CE.

**7 –** Monte Mozinho (MOZ): a large hillfort built during Roman times ([18]). Grains of rye came from a functional area at roughly 383 m a.s.l., dating to the Late Antiquity, displaying three compartments with storage structures, hearths, and a large oven ([19-21]). The archaeobotanical study revealed a considerable set of fruits/seeds, with a large amount of grains and chaff of rye. Broomcorn millet and rye are predominant. Rye grains from a storage facility in Compartment 1, and an Oven in Compartment 3 were dated, supporting two occupation phases, within Late Antiquity. Among 100 measured grains, 14 came from the storage facility, 59 from Compartment 2, and 27 from the Oven.

**8 –** Castelo de Crestuma (CRES): small site in a hill next to the river Douro at 57 m a.s.l., occupied between the Iron Age and the Medieval period ([22]). A small number of macroremains was found, most in negative structures in the highest areas of the hill. The results remain unpublished. Several rye grains were dated, revealing Late Antique ages. On other hand, the grains recovered were not enough for the biometric approach.

**9 -** Castro S. João das Arribas (CSJAMD): implanted on the edge of a cliff, at 658 m a.s.l., next to the Douro river, was initially considered an Iron Age hillfort ([23]) but recent excavations only revealed occupation between the Late Roman times and the Medieval period ([24-26]). In a central area, a large number of rye grains was collected inside two storage facilities and in their surroundings, as well as in pottery vessels ([27]). One ^14^C date from a vessel revealed a Late Antique chronology. A total of 100 grains found nearby the storage facilities (s.u. 225) were measured.

**10-** Senhora do Barrocal (SB): small settlement placed on a granitic tor at 598 m a.s.l, on very rough terrains. The occupation of SB was marked by a fire that destroyed a habitational structure, around the 10^th^-11^th^ centuries CE, being SB reoccupied afterwards, but during a short period of time ([28, 29]). A massive archaeobotanical assemblage was recovered, containing fruits/seeds, charcoal and food remains. Rye and oat were the main crops. A ^14^C date with a long interval (8^th^-10^th^ centuries CE) was obtained over a grain of rye collected in a filling of a wall. Biometric data was obtained in 100 grains from a fire level (s.u. 107), corresponding to the house´s destruction ([28, 29]).

**References**

1. Mendes-Pinto JMS. Do castro de S. Domingos a Meinedo: Proto-história e Romanização na bacia superior do rio Sousa. Oppidum. 2008; Número Especial: 45–63.
2. Nunes M, Lemos P, Leite J, Novais H. Estruturas negativas no sítio arqueológico da “Casa Romana” do Castro de São Domingos (Lousada): as fossas escavadas no saibro. In: Gutiérrez NH, Galarza JL, Hernández, RP, editors. Investigaciones arqueológicas en el valle del Duero: del Paleolítico a la Edad Media 6. Valladolid: Glyphos Publicaciones; 2018. p. 419–427.
3. Lemos P. “Casa Romana” do Castro de São Domingos (Cristelos, Lousada): escavação, estudo e musealização. In: Arnaud JM, Neves C, Martins A, editors. Arqueologia em Portugal 2020 – Estado da questão. Lisboa: Associação dos Arqueólogos Portugueses e CITCEM; 2020. p. 1277–1290. <https://doi.org/10.21747/978-989-8970-25-1/arqa93>
4. Lemos P, Nunes M, Magalhães B. A necrópole medieval do Castro de São Domingos (Lousada): resultados preliminares das campanhas de escavação 2017-2021. Oppidum. 2021; 13: 6–37.
5. Seabra L, Lemos P, Nunes M, Martín-Seijo M, Almeida R, Tereso JP. Frutos e sementes da “Casa Romana” do Castro de São Domingos (Lousada, Norte de Portugal). Al-Madan. 2023; 26 (1): 70–84.
6. Dinis AP. O povoado da Idade do Ferro do Crastoeiro (Mondim de Basto, Norte de Portugal). Braga: Cadernos de Arqueologia 13, Unidade de Arqueologia da Universidade do Minho; 2001.
7. Dinis AP, Bettencourt MAS. A Arte Atlântica do Crastoeiro (Norte de Portugal) Gallaecia. 2009; 28:41–47.
8. Seabra L, Tereso JP, Bettencourt AMS, Dinis A. Crop diversity and storage structures in the settlement of Crastoeiro (Northwest Iberia): new approaches. Trabajos Prehist. 2018; 75 (2): 361–378. <https://doi.org/10.3989/tp.2018.12221>
9. Platas I. A Memoria Final das Sondaxes Arqueolóxicas Valorativas no Castelo Pequeno de Santigoso (A Mezquita-Ourense). Pontevedra: Citania Arqueoloxía S.L., 2019. Report.
10. Pereira AS, González Celsa. Castro de Cruito. Arqueologia. 1988; 17: 151–158.
11. Pinto da Silva A. Estudo de Cinco Amostras de Macro-restos Vegetais. Oeiras: Estação Agronómica Nacional; 1990.
12. Dias LT. Tongobriga. Lisboa: Instituto Português do Património Arquitectónico (IPPAR); 1997.
13. Lima A. The Mosaics of the Church of Santa Maria Do Freixo (Marco de Canaveses, Portugal): Reflections on Its Meaning in the Context of the Late Musivaria of the Douro Valley. Journal of Mosaic Research. 2017; 10: 223–242. <https://doi.org/10.26658/jmr.357088>
14. Lima A, López-Dóriga I, Rebuge J, Pereira JA. A muralha de Tongobriga: descoberta, investigação, conservação e restauro. In: Lima A, editor. “Tongobriga”: Coletânea de Estudos comemorativos de 40 anos de Investigação. Porto: Direção Regional de Cultura do Norte - Ministério da Cultura; 2020. p. 123–177.
15. López-Dóriga I. Estudios arqueobotánicos en Tongobriga: muestreo y resultados preliminares. In: Lima A, editor. “Tongobriga”: Coletânea de Estudos comemorativos de 40 anos de Investigação. Porto: Direção Regional de Cultura do Norte - Ministério da Cultura; 2020. p. 83–97.
16. Pereira SS, Silva B, Larrazabal J, Garibo J, Nisa J, Pereira JA, Mateos R, Cosme S. A romanização no vale do Sabor: de Meirinhos a Remondes (Mogadouro). In: Dinis AP, editor. I Encontro de Arqueologia de Mogadouro. Mogadouro: Município de Mogadouro; 2014. p. 95–143.
17. Tereso JP, Pereira S, Santos F, Seabra L, Vaz FC. Cultivos de época romana no Baixo Sabor: Continuidade em tempos de mudança? In: Arnaud JM, Neves C, Martins A, editors. Arqueologia em Portugal 2020 – Estado da Questão. Lisboa: Associação dos Arqueólogos Portugueses e CITCEM; 2020. p. 1207–1220. https://doi.org/10.21747/978-989-8970-25-1/arqa87
18. Soeiro T. Monte Mozinho: apontamentos sobre a ocupação entre Sousa e Tâmega em época romana. Penafiel: Boletim Municipal de Cultura. 1984; 3º série (1): 5–323.
19. Tereso JP, Ramil-Rego P, Carvalho TP, Almeida-da-Silva R, Vaz FC. Crops and fodder: evidence for storage and processing activities in a functional area at the Roman settlement of Monte Mozinho (northern Portugal). Veget Hist Archaeobot. 2013; 22: 479–492. https://doi.org/10.1007/s00334-013-0399-x
20. Vaz FC, Tereso JP, Carvalho TP. Selection of firewood in Monte Mozinho (NW Iberia) in the Late Antiquity: A question of function and availability. Quat Int. 2017; 431, Part A: 103–115. <https://doi.org/10.1016/j.quaint.2015.10.041>
21. Vaz FC, Seabra L, Tereso JP, Carvalho TP. Combustível para um forno: Dinâmicas de ocupação de um espaço em Monte Mozinho (Penafiel) A Partir de novos dados Arqueobotânicos. In: Arnaud JM, Martins A, editors. Arqueologia em Portugal 2017 – Estado da questão. Lisboa: Associação dos Arqueólogos Portugueses; 2017. p. 1331–1345.
22. Silva AM, Gonçalves Guimarães JA, Pinto FMS, Sousa L, Leite J, Lemos P, Pereira P, Teixeira MF. O projeto castr’uíma (vila nova de gaia, 2010-2015): elementos e reflexões para um balanço prospetivo. In: Arnaud JM, Martins A, editors. Arqueologia em Portugal 2017 – Estado da questão. Lisboa: Associação dos Arqueólogos Portugueses; 2017. p. 137–154.
23. Lemos FS. O Povoamento Romano de Trás-os-Montes Oriental [PhD thesis]: University of Minho; 1993.
24. Salgado M, Pereira P. O Castro S. João das Arribas. Achegas para uma storia das Arribas. Parte I. Revista da Memória Rural. 2018; 1: 165–173.
25. Salgado M, Pereira P. O Projeto de Investigação sobre o Castro S. João das Arribas - três campanhas de escavação depois e as histórias que ainda estão por contar. Brigantia. 2018-19; XXXVI-XXXVII: 211–230.
26. Salgado M, Pereira P. O Castro S. João das Arribas. Achegas para uma storia das Arribas. Parte II. Revista da Memória Rural. 2019; 2: 225–232.
27. Seabra L, Pereira P, Salgado M, Martín-Seijo M, Almeida-da-Silva R, Tereso JP. Crops on the edge of a cliff: Storage at Castro S. João das Arribas (Northwest Iberia) in the Late Antiquity. J Archaeol Sci: Reports. 2022; 44: 103528. <https://doi.org/10.1016/j.jasrep.2022.103528>
28. Tente C. No smoke without fire. Burning and changing settlements in 10th-century central-northern Portugal. In: Brady N, Theune C, editors. Settlement Change across Medieval Europe. Old Paradigms and New Vistas. Leiden: Sidestone Press; 2019. p. 395–403.
29. Seabra L, Tente C, Vaz FC, Oliveira C, González Carretero L, Tereso JP. Crops on the Rocks: Production, Processing, and Storage at the Early Medieval Site of Senhora Do Barrocal (Municipality of Sátão, Central Portugal). Plants. 2022; 11: 471. https://doi.org/10.3390/plants11040471
