# Supplementary material for: The introduction and spread of rye (Secale cereale) in the Iberian Peninsula
Source: PLoS One. 2023 May 10;18(5):e0284222. doi: 10.1371/journal.pone.0284222 (PMC10171662; doi:10.1371/journal.pone.0284222)
Supplement: S2 Text — (DOCX) [file pone.0284222.s006.docx]

**Supporting Information (S4 Text)**

**Oxcal code (CQL2) used to create the multiphase model.**

Plot()

{

Sequence("Rye chronology")

{

Boundary("Iron Age/Early Roman start")

{

color="green";

};

KDE_Plot ("Iron Age/Early Roman")

{

R_Date("TONG-Circular house", 2158, 21);

R_Date("CDCR-Pit 51", 2139, 20);

R_Date("CRT-Pit 18.1", 2132, 31);

R_Date("CPS-Utilization layer", 2120, 30);

R_Date("TONG-Wall", 2062, 22);

R_Date("CRUI-Floor", 2050, 30);

R_Date("CRT-Pit 18.2", 2027, 25);

color=“blue"

};

Boundary("Iron Age/Early Roman end")

{

color="red";

};

Interval("Iron Age/Early Roman-Roman/Late Antiquity");

Boundary("Roman/Late Antiquity start")

{

color="green";

};

KDE_Plot ("Roman/Late Antiquity ")

{

R_Date("CDG-Oven", 1819, 23);

R_Date("MOZ-Quad. structure", 1770, 30);

R_Date("TONG-Impluvium house", 1692, 27);

R_Date("MOZ-Oven", 1640, 30);

R_Date("CRES-A1_1003", 1607, 26);

R_Date("CRES-A1_469", 1557, 24);

R_Date("CRES-A1_1010", 1538, 24);

R_Date("CRES-E_728", 1501, 24);

R_Date("CRES-E_734", 1471, 28);

R_Date("CSJAMD-Vessel", 1435, 24);

color=“blue"

};

Boundary("Roman/Late Antiquity end")

{

color="red";

};

};

};
